# Melatonin May Improve Post-Thaw Sperm Motility in *Epinephelus fuscoguttatus* by Potentially Regulating Mitochondrial mPTP via the MT2/PI3K/GSK-3β Pathway: First Evidence in Teleosts

**DOI:** 10.3390/antiox15040422

**Published:** 2026-03-27

**Authors:** Yuxin Zhang, Qingxin Ruan, Weiwei Zhang, Yingxin Wu, Jiajie Li, Qinghua Wang, Fanming Guo, Yang Yang, Zining Meng

**Affiliations:** 1 School of Life Sciences, State Key Laboratory of Biocontrol/Southern Marine Science and Engineering Guangdong Laboratory (Zhuhai)/Guangdong Provincial Key Laboratory of Aquatic Economic Animals, Sun Yat-sen University, Guangzhou 510275, China; zhangyx558@mail2.sysu.edu.cn (Y.Z.); ruanqx3@mail2.sysu.edu.cn (Q.R.); zhangww79@mail2.sysu.edu.cn (W.Z.); wuyx328@mail2.sysu.edu.cn (Y.W.); lijj379@mail2.sysu.edu.cn (J.L.); wangqh55@mail2.sysu.edu.cn (Q.W.); guofm@mail2.sysu.edu.cn (F.G.); 2 Key Laboratory of Tropical Marine Fish Germplasm Innovation and Utilization, Ministry of Agriculture and Rural Affairs, Hainan Engineering Research Center for Germplasm Innovation and Utilization, Hainan Chenhai Aquatic Co., Ltd., Sanya 570000, China; 3 China-ASEAN Belt and Road Joint Laboratory on Mariculture Technology, Guangzhou 510275, China

**Keywords:** *Epinephelus fuscoguttatus*, melatonin, sperm cryopreservation, mitochondrial function, MT2 receptor, PI3K/Akt/GSK-3β signaling pathway, reactive oxygen species

## Abstract

Melatonin, a well-known antioxidant, has been widely used in sperm cryopreservation of various animals, but its regulatory mechanism in fish remains unclear. This first study on teleosts suggests a potential molecular mechanism by which melatonin may improve post-thaw sperm quality of *Epinephelus fuscoguttatus* via targeting mitochondrial function. Compared with the melatonin group, the MT1 receptor-inhibited group showed slightly higher sperm motility (77.09 ± 3.41% vs. 76.50 ± 1.10%), significantly inhibited mitochondrial permeability transition pore (mPTP) opening (12.64 ± 1.05% vs. 18.29 ± 1.38%), and maintained higher mitochondrial membrane potential (MMP; 85.86 ± 0.18% vs. 81.81 ± 0.69%), with both groups performing better than the control. In contrast, the MT2-inhibited and MT1/2 dual-inhibited groups exhibited reduced sperm quality compared with the MT group, suggesting that MT2 may serve as the core receptor for melatonin to regulate mitochondrial homeostasis in teleosts. Mechanistically, melatonin-activated MT2 potentially inhibits mPTP opening via the PI3K/Akt/GSK-3β pathway, and this protective effect was abrogated by the PI3K and GSK-3β inhibitors. This receptor-mediated process synergized with melatonin’s direct antioxidant effect, as ROS levels in all melatonin-treated groups were significantly lower than the control. This study is the first to find pharmacological evidence for the melatonin–MT2/PI3K/GSK-3β axis in maintaining teleost sperm mitochondrial function; it also reveals potential mechanistic differences between teleosts and mammals and fills a critical knowledge gap regarding this signaling cascade in teleost reproductive biology.

## 1. Introduction

Brown-marbled grouper (*Epinephelus fuscoguttatus*) is a warm-water reef fish widely distributed in the Indo-Pacific region [1]. Due to overfishing driven by export market demand, wild populations have declined significantly, leading to its listing on the IUCN Red List of Threatened Species [2]. To meet market demand, brown-marbled grouper is extensively cultured in China and Southeast Asia. However, as a typical protogynous hermaphrodite, aquaculture often faces a shortage of male broodstock gametes. Consequently, sperm cryopreservation is vital for preserving germplasm resources, ensuring gamete supply, and supporting conservation efforts for this endangered grouper species [3]. Despite its advantages, the freeze–thaw process inflicts substantial damage on spermatozoa, impairing their motility, functionality, and fertilizing capacity [4]. Notably, oxidative stress induced by the accumulation of reactive oxygen species (ROS) is a key factor contributing to this post-thaw quality deterioration [5,6]. Therefore, supplementing cryoprotectants with exogenous antioxidants represents a critical strategy to mitigate such cryoinjury [7]. Currently, the sperm cryopreservation system for brown-marbled grouper mainly relies on traditional permeating cryoprotectants such as dimethyl sulfoxide (DMSO) and glycerol, with antioxidant components limited to single molecules like vitamin C and glutathione (GSH) [5,8]. Although such systems can partially alleviate cell dehydration and ice crystal damage caused by low temperatures, their efficacy in mitigating mitochondrial dysfunction induced by excessive reactive oxygen species (ROS) during freeze–thaw—including lipid peroxidation, protein oxidation, mitochondrial membrane potential (∆Ψm) collapse, and abnormal opening of the mitochondrial permeability transition pore (mPTP)—is limited [9,10]. Therefore, there is an urgent need to explore novel high-efficiency antioxidants with multi-target effects (direct antioxidation, mitochondrial protection, and apoptosis regulation), clarify their protective mechanisms, and optimize the cryopreservation protocol. This will provide technical support for germplasm conservation and large-scale seedling production of this species [11,12].

Melatonin, a lipophilic indoleamine hormone primarily secreted by the pineal gland, is well recognized for its potent antioxidant properties and key role in regulating circadian rhythms [13]. In recent years, its function in germ cell cryopreservation has garnered increasing attention [14]. Studies have demonstrated that melatonin not only effectively scavenges ROS and mitigates oxidative damage but also modulates signaling pathways associated with cell apoptosis and survival—processes crucial for preserving the structural and functional integrity of sperm [15]. Substantial evidence confirms that supplementing cryoprotectants with an optimal concentration of melatonin significantly improves post-thaw sperm motility, plasma membrane integrity, acrosome integrity, and mitochondrial function while reducing levels of malondialdehyde (MDA), a biomarker of lipid peroxidation. Ultimately, this enhancement of sperm quality translates to improved fertilizing capacity across diverse species, including cattle, sheep, fish, and humans [16,17,18,19]. Notably, melatonin exhibits a strong mitochondrial-targeting property, which serves as the key basis for its protective effects on germ cells. It can be actively transported into mitochondria via the oligopeptide transporters PEPT1/2 located on the mitochondrial membrane, and even synthesized locally within mitochondria through the key enzymes AANAT and ASMT, ensuring high-concentration enrichment inside mitochondria. This allows melatonin to directly neutralize reactive oxygen species (ROS) and stabilize mitochondrial membrane potential (∆Ψm) [20,21]. This mitochondrial enrichment characteristic endows melatonin with distinct advantages over traditional antioxidants in alleviating freeze–thaw-induced mitochondrial dysfunction [8,20]. Although melatonin exerts significant protective effects on sperm cryopreservation in various animals [19], its precise underlying mechanisms—especially the detailed regulation of relevant signaling pathways—remain poorly understood, with in-depth research still lacking in fish sperm cryopreservation regarding melatonin receptor-mediated signaling pathways and the molecular mechanisms of its mitochondrial function modulation.

It is well established that the protective effect of melatonin on cryopreserved sperm in mammals is mediated primarily through the MT1 receptor [22,23]. Furthermore, the melatonin–MT1/PI3K/GSK-3β axis constitutes a critical pathway for maintaining mitochondrial function in goats [24]. In mammals, melatonin exerts its biological functions through two primary pathways: receptor-mediated and non-receptor-mediated. In the receptor-mediated pathway, melatonin binds to G protein-coupled receptors on sperm and reproductive cells. This interaction regulates the cAMP/PKA signaling pathway, suppresses the expression of pro-apoptotic genes, and enhances the expression of the anti-apoptotic gene *Bcl-2*. Concurrently, melatonin activates the RORα receptor to modulate the expression of genes related to steroid synthesis, optimizing the sperm microenvironment [25,26]. In contrast, the non-receptor-mediated pathway primarily relies on melatonin’s potent intrinsic antioxidant capacity to directly scavenge ROS and enhance the activity of endogenous antioxidant enzymes [27], thereby reinforcing the sperm’s antioxidant defense system [28]. To date, systematic studies on the functional differentiation of melatonin receptors MT1 and MT2 in fish germ cells remain scarce. Multiple melatonin receptor subtypes have been identified in teleosts and are expressed in various tissues including the brain, pituitary, and gonads [29,30], yet their expression patterns and specific functions in sperm have not been fully elucidated. Moreover, in mammals, melatonin is well known to regulate cellular metabolism and antioxidant responses via G protein-coupled signaling pathways mediated by MT1/MT2 receptors [29,31,32], but the evolutionary conservation of these canonical receptor signaling pathways in teleosts lacks systematic pharmacological validation. Meanwhile, the mitochondrial permeability transition pore (mPTP) is recognized as a crucial target for maintaining mitochondrial homeostasis, but no study has directly demonstrated that it serves as a downstream effector of melatonin receptor signaling pathways in fish sperm. Therefore, clarifying the roles of MT1/MT2 receptors in fish sperm and their association with mitochondrial function represents an important research direction for understanding the protective mechanisms of melatonin.

Building upon recent findings from our laboratory on grouper sperm cryopreservation [33,34], the present study focused on MT1/2 receptors and their downstream signaling pathways. Using exogenously administered specific inhibitors and complementary experimental approaches, we investigated whether melatonin improves post-thaw sperm quality by activating MT receptors, subsequently regulating the PI3K/Akt/GSK3β signaling axis and modulating mitochondrial permeability transition pore (mPTP) opening. This research aims to explore the potential molecular mechanism by which melatonin mitigates oxidative damage in fish sperm through the MT1/2-PI3K/Akt/GSK-3β-mPTP pathway, providing a theoretical basis for developing MT2/PI3K-targeted cryoprotective strategies.

## 2. Materials and Methods

### 2.1. Broodstock Rearing and Gamete Collection

Sexually mature male *Epinephelus fuscoguttatus* broodstock (9–10 years old; body weight 7 ± 1 kg) were obtained from the Gancheng Base of Hainan Chenghai Aquatic Products Co., Ltd. (Sanya, China) (18.818° N, 108.633° E). During the natural reproductive season (August to October 2024), the fish were maintained in outdoor concrete tanks (6 × 10 × 2 m) under a natural photoperiod (14 L:10 D) and temperature (26–28 °C) with flow-through seawater. Broodstock were fed commercial feed every 48 h according to standard enterprise protocols, and residual feed was removed immediately after feeding. Semen collection was performed by gentle abdominal compression after manually drying the urogenital region to avoid contamination by excreta. Samples were stored at 4 °C, and initial motility assessment and cryopreservation procedures were initiated within 30 min post-collection. A total of 3 sexually mature males with initial sperm motility ≥ 90% were used in this study, and semen from these males was pooled to ensure a sufficient sample volume for all of the experimental groups and eliminate individual differences among broodstock. All animal handling procedures were approved by the Institutional Animal Care and Use Committee of Sun Yat-sen University.

### 2.2. Experimental Design

To elucidate the molecular mechanism underlying melatonin-mediated protection against oxidative stress in post-thaw *Epinephelus fuscoguttatus* sperm, exogenous specific inhibitors were employed in the present study. We hypothesized that melatonin activates the PI3K/Akt/GSK-3β signaling pathway via MT1/MT2 receptors, thereby inhibiting excessive mitochondrial permeability transition pore (mPTP) opening, stabilizing mitochondrial membrane potential (MMP), improving sperm motility, and reducing reactive oxygen species (ROS) levels. All experimental reagents were prepared on the basis of a basic diluent (0.3 M glucose, 10% DMSO). The working concentrations of these inhibitors were selected based on previous reports in sperm cryopreservation studies and further optimized by preliminary experiments [24]. The detailed information of specific inhibitors used in this study is shown as follows, and all of the experimental groups were formulated by supplementing 0.1 mg/mL melatonin and corresponding inhibitors to the basic diluent:

#### 2.2.1. Inhibitor Information

The detailed information of these inhibitors, including their targets and working concentrations, is summarized in Table 1.

#### 2.2.2. Experimental Grouping

According to the above inhibitor treatment, a total of 8 experimental groups were set up in this study, with the specific formulation as follows:

Control: Basic diluent only;
Melatonin (MT): Basic diluent + 0.1 mg/mL melatonin;
MT1^−^: Basic diluent + 0.1 mg/mL melatonin + 8-M-PDOT (10^−6^ M);
MT2^−^: Basic diluent + 0.1 mg/mL melatonin + 4-P-PDOT (10^−6^ M);
MT1/2^−^: Basic diluent + 0.1 mg/mL melatonin + Luzindole (10^−6^ M);
PI3K^−^: Basic diluent + 0.1 mg/mL melatonin + LY294002 (10^−5^ M);
GSK-3β^−^: Basic diluent + 0.1 mg/mL melatonin + SB415286 (10^−5^ M);
PI3K/GSK-3β^−^: Basic diluent + 0.1 mg/mL melatonin + LY294002 (10^−5^ M) + SB415286 (10^−5^ M).

### 2.3. Sperm Cryopreservation and Thawing

**Figure 1:**
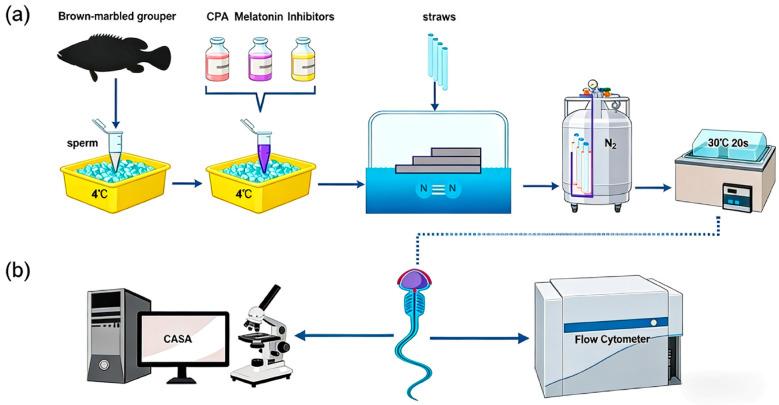
Schematic diagram of sperm cryopreservation procedure and detection indicators: (**a**) Steps of semen collection and cryopreservation: After semen collection, the sperm were mixed with basic diluent, 0.1 mg/mL melatonin and different inhibitors (8-M-PDOT: 10^−6^ M; 4-P-PDOT: 10^−6^ M; Luzindole: 10^−6^ M; LY294002: 10^−5^ M; SB415286: 10^−5^ M), and aliquoted into 0.25 mL straws. Subsequently, the samples were fumigated at a certain height above the liquid nitrogen surface, and then immersed in liquid nitrogen for long-term storage. For use, the samples were thawed in a 30 °C water bath for 20 s and then placed on ice for subsequent use. (**b**) Detection of sperm motility, reactive oxygen species (ROS) levels, as well as mitochondrial membrane potential (MMP) and mitochondrial permeability transition pore (mPTP) levels was performed using a computer-assisted sperm analysis (CASA) system and flow cytometry within 30 min post-thawing. All inhibitors were equilibrated with sperm samples at 4 °C for 10 min before cryopreservation.

Cryopreservation was performed using a slow-cooling method in a foam box [35]. Pooled semen (motility > 90%) was diluted at a ratio of 1:3 (semen:diluent) with treatment-specific diluents, equilibrated at 4 °C for 10 min, loaded into 0.25 mL straws (IMV, L’Aigle, France), and placed horizontally on a floating plate 3 cm above liquid nitrogen for 10 min before immersion in liquid nitrogen. For thawing, straws were transferred to a 30 °C water bath for 20 s (Figure 1a). Post-thaw samples were held on crushed ice (4 °C) until analysis (Figure 1b). Three replicate cryopreservations were performed for each treatment. All replicates were technical replicates from the same pooled semen sample.

### 2.4. Detection of Post-Thaw Sperm Quality

#### 2.4.1. Sperm Motility

Motility was assessed using a Computer-Assisted Sperm Analysis (CASA) system with the following settings: 30 frames per second (fps); sperm head area 1–90 μm^2^; motile sperm defined as curvilinear velocity (VCL) > 10 μm/s. Fresh semen (1 μL) was activated in 99 μL filtered seawater (0.22 μm) and analyzed within 3 s. The thawed samples (1 μL) were activated in 29 μL filtered seawater and analyzed within 5–10 s. Only samples with initial motility > 90% were used for subsequent experiments.

#### 2.4.2. ROS Detection

Intracellular ROS levels were quantified using the ROS Assay Kit (Beyotime, Shanghai, China). Samples were incubated with 1 μM DCFH-DA for 20 min at 25 °C in the dark. Positive controls were treated with 1 μL Rosup. After washing three times with phosphate-buffered saline (PBS), samples were analyzed via flow cytometry.

#### 2.4.3. mPTP Detection

mPTP opening was assessed using the mPTP Assay Kit (Beyotime, Shanghai, China). Samples were washed three times with PBS and incubated with Calcein AM staining solution (negative control), quenching solution (experimental groups), or Ionomycin (positive control) for 30 min at 25 °C in the dark. After three additional washes with PBS, flow cytometry was performed.

#### 2.4.4. MMP Detection

Mitochondrial membrane potential (MMP) was evaluated using the MMP Assay Kit (Beyotime, Shanghai, China). Samples were incubated with JC-1 staining solution (2.5 μL/10^7^ cells) at 37 °C for 20 min in the dark, washed twice with PBS, resuspended in assay buffer, and analyzed using flow cytometry.

#### 2.4.5. Flow Cytometry

ROS, mPTP, and MMP levels were analyzed using a CytoFlex flow cytometer (Beckman Coulter, Brea, CA, USA). A 488 nm laser was used to excite fluorophores, with emissions collected at 525/40 nm (green fluorescence) and 585/42 nm (red fluorescence). For each sample, ≥10,000 events were recorded at a rate of 300–500 cells/s. Data were processed using CytExpert software (version 2.5.0.77, Beckman Coulter, Brea, CA, USA).

### 2.5. Statistical Analysis

Data are presented as mean ± standard deviation (SD). Percentage data were subjected to arcsine transformation before analysis. Normality of data was verified prior to ANOVA. The differences among multiple groups were assessed by one-way analysis of variance (ANOVA) followed by Tukey’s post hoc test, while the differences between two groups were analyzed using *t*-test. Statistical significance was set at *p* < 0.05. All analyses were performed using SPSS 20.0 software (SPSS, Chicago, IL, USA).

## 3. Results

### 3.1. Results of Sperm Motility Assay

**Figure 2:**
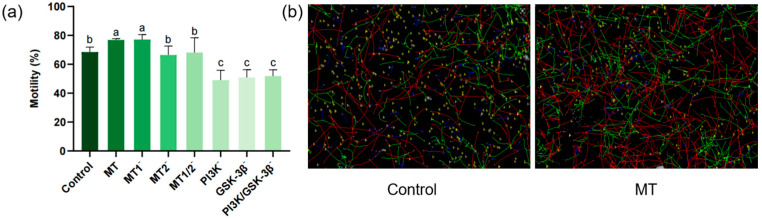
Effects of different treatments on post-thaw sperm motility and movement trajectories (*n* = 3): (**a**) Bar chart of the effects of different inhibitors (8-M-PDOT: 10^−6^ M; 4-P-PDOT: 10^−6^ M; Luzindole: 10^−6^ M; LY294002: 10^−5^ M; SB415286: 10^−5^ M) on post-thaw sperm motility: “−” indicates the inhibition of corresponding receptors/pathways; different superscript letters indicate significant differences (*p* < 0.05). (**b**) Left: Sperm movement trajectory of the control group. Right: Sperm movement trajectory of the MT group (0.1 mg/mL). Red lines: Rapid progressive sperm. Green lines: Slow progressive sperm. Blue lines: Non-progressive sperm. Note: All inhibitors were equilibrated with sperm samples at 4 °C for 10 min before cryopreservation.

As shown in Figure 2a, the post-thaw sperm motility in the MT group (76.50 ± 1.10%) was significantly higher than that in the control group (68.45 ± 8.44%). No significant difference was observed in post-thaw sperm motility between the MT2^−^ group (66.43 ± 6.16%) and the MT1/2^−^ group (68.10 ± 10.24%), while both were significantly lower than the MT group and the MT1^−^ group (77.09 ± 3.41%). There was no significant difference in sperm motility between the MT group and the MT1^−^ group. No significant differences were detected in post-thaw sperm motility among the PI3K^−^ group (49.03 ± 6.84%), GSK-3β^−^ group (50.86 ± 5.40%) and PI3K/GSK-3β^−^ group (51.80 ± 4.42%), whereas all these groups exhibited significantly lower motility compared with the MT group.

### 3.2. Determination of Reactive Oxygen Species (ROS) Production

**Figure 3:**
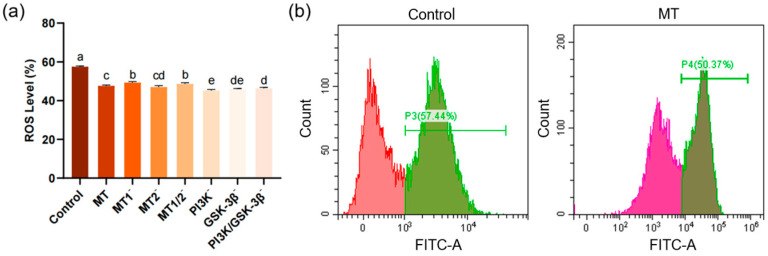
Bar chart and flow cytometry plots showing the effects on post-thaw sperm ROS levels (*n* = 3): (**a**) Bar chart of the effects of different inhibitors (8-M-PDOT: 10^−6^ M; 4-P-PDOT: 10^−6^ M; Luzindole: 10^−6^ M; LY294002: 10^−5^ M; SB415286: 10^−5^ M) on post-thaw sperm ROS levels, “−” indicates inhibition of corresponding receptors or pathways, different superscript letters indicate significant differences (*p* < 0.05). (**b**) Left: Flow cytometry histogram of the control group with the abscissa representing FITC fluorescence intensity. Right: Flow cytometry histogram of the MT group (0.1 mg/mL) with the abscissa representing FITC fluorescence intensity. Green FITC fluorescence intensity (indicating intracellular ROS level). All inhibitors were equilibrated with sperm samples at 4 °C for 10 min before cryopreservation.

The ROS level in the MT group (47.61 ± 0.47%) was significantly lower than that in the control group (57.48 ± 0.38%). Although ROS levels fluctuated in other experimental groups, all were significantly lower than that in the control group (Figure 3a).

### 3.3. Results of Mitochondrial Permeability Transition Pore (mPTP) Assay

**Figure 4:**
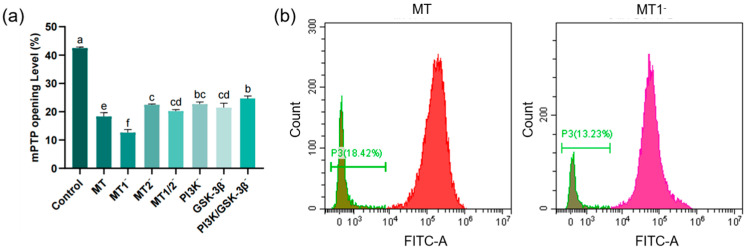
Bar chart and flow cytometry plots showing post-thaw sperm mPTP opening levels (*n* = 3): (**a**) Bar chart of the effects of different inhibitors (8-M-PDOT: 10^−6^ M; 4-P-PDOT: 10^−6^ M; Luzindole: 10^−6^ M; LY294002: 10^−5^ M; SB415286: 10^−5^ M) on post-thaw sperm mPTP opening levels, “−” indicates inhibition of corresponding receptors or pathways, different superscript letters indicate significant differences (*p* < 0.05). (**b**) Left: Flow cytometry histogram of the MT group (0.1 mg/mL) with the abscissa representing FITC fluorescence intensity. Right: Flow cytometry histogram of the MT1^−^ group with the abscissa representing FITC fluorescence intensity. Green FITC fluorescence intensity (indicating mPTP opening level). All inhibitors were equilibrated with sperm samples at 4 °C for 10 min before cryopreservation.

As shown in Figure 4a, the mPTP opening level in the MT group (18.29 ± 1.38%) was significantly reduced compared with the control group (42.46 ± 0.37%). The mPTP opening level in the MT group was significantly lower than that in the MT2^−^ group (22.45 ± 0.26%), but significantly higher than that in the MT1^−^ group (12.64 ± 1.05%). No significant difference was found in mPTP opening level between the MT2^−^ group and the MT1/2^−^ group (20.20 ± 0.52%). There was no significant difference in mPTP opening level between the PI3K^−^ group (22.68 ± 0.65%) and the PI3K/GSK-3β^−^ group (21.44 ± 0.56%), and both were significantly higher than that in the MT group.

### 3.4. Results of Mitochondrial Membrane Potential (MMP) Assay

**Figure 5:**
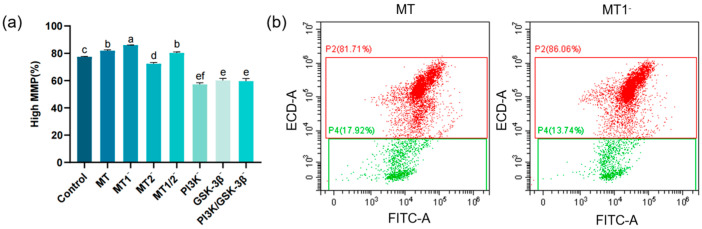
Bar chart and flow cytometry plots showing the effects on post-thaw sperm MMP levels (*n* = 3): (**a**) Bar chart of the effects of different inhibitors (8-M-PDOT: 10^−6^ M; 4-P-PDOT: 10^−6^ M; Luzindole: 10^−6^ M; LY294002: 10^−5^ M; SB415286: 10^−5^ M) on post-thaw sperm MMP levels, “−” indicates inhibition of corresponding receptors or pathways, different superscript letters indicate significant differences (*p* < 0.05). (**b**) Left: Flow cytometry histogram of the MT group (0.1 mg/mL) with the abscissa representing FITC fluorescence intensity. Right: Flow cytometry histogram of the MT1^−^ group with the abscissa representing FITC fluorescence intensity. All inhibitors were equilibrated with sperm samples at 4 °C for 10 min before cryopreservation.

The MMP level in the MT group (81.81 ± 0.69%) was significantly elevated compared with the control group (77.37 ± 0.34%). The MMP level in the MT group was significantly lower than that in the MT1^−^ group (85.86 ± 0.18%) but significantly higher than that in the MT2^−^ group (72.29 ± 0.90%). No significant difference was observed in MMP level between the MT group and the MT1/2^−^ group (80.22 ± 0.78%). No significant differences were detected in MMP levels among the PI3K^−^ group (57.20 ± 1.16%), GSK-3β^−^ group (60.10 ± 1.51%) and PI3K/GSK-3β^−^ group (59.59 ± 2.00%), and all these groups showed significantly lower MMP levels than the MT group (Figure 5a).

## 4. Discussion

The present study suggested that melatonin, a highly efficient antioxidant, significantly improved the comprehensive quality of post-thaw sperm in the cryopreservation system for brown-marbled grouper sperm. Its protective effects were mainly reflected in the effective scavenging of reactive oxygen species (ROS), alleviation of oxidative damage [36], and inhibition of mitochondrial permeability transition pore (mPTP) opening. These effects synergistically stabilized the mitochondrial membrane potential (MMP) and ultimately enhanced the motile performance of post-thaw sperm, which is consistent with the findings of studies on other fish models [18,33,37]. The antioxidant mechanisms of melatonin are multidimensional. First, its molecular structure endows it with strong direct free radical-scavenging capacity [38]. Second, it can significantly upregulate the activities of endogenous antioxidant enzymes, including superoxide dismutase (SOD), catalase (CAT), and glutathione peroxidase (GPx) [39]. Additionally, its metabolites (e.g., AFMK) also contribute to a synergistic antioxidant effect [40]. Collectively, these mechanisms construct a robust antioxidant defense network that effectively mitigates oxidative stress-induced damage to sperm during the freeze–thaw process [41].

Notably, the mechanism underlying melatonin’s protective effect against oxidative damage in post-thaw brown-marbled grouper sperm is not limited to receptor-mediated signaling pathways. Owing to its intrinsic lipophilic property, melatonin can freely penetrate the sperm plasma membrane [42] and directly neutralize hydroxyl radicals, superoxide anions, and other reactive species via electron donors on its indole ring [15]. Key experimental evidence supports this non-receptor-dependent pathway: although the MT2-specific antagonist (4-P-PDOT) or PI3K pathway inhibitor (LY294002) partially attenuated the antioxidant effect of melatonin, the ROS levels in post-thaw sperm of all melatonin-treated experimental groups remained significantly lower than those in the control group. This suggests that its ROS-scavenging effect may not be entirely dependent on receptor binding or the activation of downstream signaling pathways. This direct antioxidant capacity is particularly crucial during the initial burst of oxidative stress in the early freeze–thaw stage, when the receptor and pathway systems may not be fully activated. At this stage, melatonin can timely scavenge excess ROS, laying a foundation for its subsequently activated receptor signaling mechanism; together, they maintain the redox homeostasis of sperm [43]. Such a dual antioxidant mode is the key reason why melatonin exerts a more stable protective effect than single-pathway antioxidants in the cryopreservation of fish sperm.

As a key organelle for sperm energy metabolism, mitochondrial integrity exerts a decisive effect on sperm quality and is the core determinant of post-thaw sperm motility [44,45]. Excessive ROS generated during the freeze–thaw process is a critical factor inducing the abnormal opening of mPTP [46,47]. In previously reported studies on mammals and vertebrates, excessive mPTP opening has been demonstrated to trigger mitochondrial matrix swelling, mitochondrial membrane potential (MMP) collapse and cytochrome c (Cytc) release, which in turn activates the caspase cascade and ultimately induces apoptosis [47]. These molecular events have been well-characterized in existing reproductive and mitochondrial biology research. This process has been shown to severely impair sperm ATP synthesis, motile capacity and fertilization potential [20,48]. However, the present study did not directly detect Cytc release, caspase activity or apoptosis-related markers, and the inferences regarding the associated apoptotic processes are solely based solely on the phenotypic changes in mPTP and MMP in this study as well as well-documented evidence from existing studies [20,47]. In fish, sperm motility is primarily dependent on ATP produced via mitochondrial oxidative phosphorylation [11]. During cryopreservation and thawing, excessive reactive oxygen species (ROS) can induce the abnormal opening of the mitochondrial permeability transition pore (mPTP), leading to a rapid decrease in mitochondrial membrane potential (MMP). Sustained opening of mPTP not only disrupts the electrochemical gradient across the mitochondrial inner membrane but also inhibits ATP production and activates apoptosis-related signaling pathways [49,50]. This process is considered a major mechanism underlying the decreased motility of sperm after freezing–thawing [1]. In the present study, melatonin significantly inhibited the abnormal opening of mPTP and maintained the stability of MMP in brown-marbled grouper sperm, suggesting that melatonin promotes ATP synthesis and thereby improves sperm motility. These results indicate that mitochondrial protection may be a crucial component underlying the antioxidant effects of melatonin. Therefore, the regulation of mPTP opening status remains a key target in research on the protection of cryopreserved sperm [51].

This study is the first to provide pharmacological evidence that melatonin may regulate mitochondrial function mainly via the MT2 receptor in post-thaw brown-marbled grouper sperm. Key experimental evidence showed that the group treated with 8-M-PDOT (a specific activator of the MT2 receptor) effectively inhibited mPTP opening, with an effect comparable to that of the MT group. In contrast, the 4-P-PDOT group (MT2 receptor inhibitor) or the Luzindole group (MT1/MT2 dual antagonist) exhibited significantly higher mPTP opening levels than the MT group. Combined with the decreased MMP and reduced sperm motility accompanied by MT2 inhibition, this finding suggests that MT2 may act as the major functional receptor for melatonin in maintaining sperm mitochondrial homeostasis in this species. This receptor preference has important species-specific implications and stands in sharp contrast to the MT1-dominated mode commonly reported in mammalian sperm [23,24,52], suggesting that teleosts may have evolved unique functional differentiation of melatonin receptors during evolution [53]. We speculate that the MT2 receptor in *E. fuscoguttatus* sperm may have advantages in subcellular localization, expression abundance, or signal coupling specificity; however, the species specificity of receptor antagonists and their exact mechanisms remain to be further investigated. The expression levels and subcellular localization of MT1 and MT2 receptors may vary significantly across different tissues, and such differences may lead to the functional differentiation of these two receptors under physiological conditions. In mammalian sperm, MT1 and MT2 receptors have been detected in the sperm head and midpiece regions and are involved in the regulation of sperm metabolism and motility [32,54]. For the brown-marbled grouper, the expression abundance and subcellular localization of MT1 and MT2 receptors in sperm remain to be further explored. If the MT2 receptor is abundantly distributed and enriched in sperm mitochondria, it may directly regulate mitochondrial function through specific signal transduction pathways, thereby explaining its functional advantages in antioxidant protection. In the future, immunofluorescence localization and Western blotting can be used to detect the expression and distribution of MT1/MT2 receptors in grouper sperm, so as to further verify the functional differentiation of melatonin receptors in different species.

Further investigations revealed that the protective effect of melatonin on sperm mitochondria mediated by the MT2 receptor may be dependent on the potential activation of the downstream PI3K/Akt/GSK-3β signaling pathway, and the present study only employed pharmacological inhibitors to investigate the involvement of this pathway without conducting direct validation of the key molecular indicators for pathway activation (e.g., Akt phosphorylation, GSK-3β Ser9 phosphorylation). Mechanistic studies indicated that the effects of melatonin on inhibiting mPTP opening and stabilizing MMP were abrogated when this pathway was blocked by the PI3K inhibitor (LY294002) or GSK-3β inhibitor (SB415286). The PI3K/Akt/GSK-3β pathway is a core signaling hub regulating cell survival, metabolism, and stress responses [55]. In previously reported signal transduction studies, the activation of PI3K has been confirmed to lead to the phosphorylation and activation of Akt (protein kinase B), which in turn phosphorylates and inhibits its key downstream target, GSK-3β [56]. This is crucial because activated GSK-3β has been identified as a key factor promoting mPTP opening [57,58]. Through Akt-mediated inhibition of GSK-3β activity, melatonin signaling is suggested to effectively block the pro-opening effect of GSK-3β on mPTP components, thereby stabilizing the mitochondrial membrane structure and preventing the abnormal opening of mPTP [59,60]. Notably, the conclusion that “melatonin may activate the PI3K/Akt/GSK-3β pathway” in the present study is solely based on the phenotypic analysis of pharmacological inhibitors rather than direct molecular-level validation, and the specific molecular events underlying pathway activation remain to be elucidated. The PI3K/Akt/GSK-3β signaling pathway plays critical roles in regulating cellular metabolism, antioxidant responses, and mitochondrial function. The present study demonstrated that inhibition of the PI3K/Akt/GSK-3β pathway not only weakened the protective effect of melatonin on brown-marbled grouper sperm, but also significantly reduced the basic motility of sperm, indicating that this pathway is not only an important downstream effector of melatonin signaling, but also a central regulatory pathway for maintaining the normal physiological function of sperm. Similar mechanisms have been confirmed in mammalian sperm studies, in which PI3K/Akt signaling improves sperm motility by regulating mitochondrial function and cell survival signals [61], and melatonin has also been shown to protect sperm from oxidative stress through the PI3K/Akt signaling pathway. This suggests that the PI3K/Akt/GSK-3β pathway is evolutionarily conserved in regulating sperm energy metabolism and mitochondrial homeostasis [62]. In addition, clinical studies of melatonin in neurodegenerative diseases have shown that it has high safety and a clear dose range, which provides a reference for its application in aquaculture [63]. Future studies should further optimize the concentration, treatment duration, and combined application strategies of melatonin or MT2 agonists in sperm cryopreservation media to achieve the optimal protective effect.

The functional importance of this signaling cascade was also pharmacologically verified in the assessment of sperm motility: treatment with pathway inhibitors not only counteracted the protective effect of melatonin but also resulted in motile parameters lower than those in the control group. This decreasing trend may be attributed to the impairment of basic sperm metabolic functions by the inhibitors themselves [64] or their potential cytotoxicity, further highlighting the fundamental role of the PI3K/Akt/GSK-3β pathway in maintaining the normal physiological functions of sperm. The PI3K/Akt/GSK-3β pathway is highly conserved in the regulation of cell survival and mitochondrial function [65,66]. The present study is the first to provide pharmacological evidence for its potential core role in fish sperm cryoprotection: upon binding to the MT2 receptor, melatonin may activate the PI3K/Akt/GSK-3β cascade, particularly by potentially inhibiting the pro-mPTP opening activity of GSK-3β. This process may effectively prevent excessive mPTP opening, thereby maintaining MMP stability, ensuring efficient ATP supply, and ultimately enhancing the motile capacity of post-thaw sperm.

It should be noted that in practical aquaculture production, sperm motility does not always equate to fertilization success, and sperm motility and fertilization capacity are not necessarily positively correlated [7]. Although our previous research has confirmed that 0.1–0.25 mg/mL melatonin can significantly improve the motility and fertilization capacity of frozen–thawed brown-marbled grouper sperm [33], the present study, which focuses on mechanistic elucidation, did not further conduct a systematic evaluation of fertilization and hatching-related indicators. Based on the MT2 receptor/PI3K/Akt/GSK-3β pathway clarified in this study, highly selective receptor agonists or pathway modulators can be developed in a targeted manner in the future to further enhance the protective effects of melatonin, improve the quality and fertilization potential of frozen sperm, and thereby better serve germplasm resource conservation and aquatic breeding practices, facilitating the translation of basic research findings into practical production applications. In addition, this regulatory mechanism can also provide novel insights and theoretical references for the cryopreservation of gametes in other aquatic organisms and even mammals, indicating that relevant research directions still hold important research value and broad development prospects.

## 5. Conclusions

**Figure 6:**
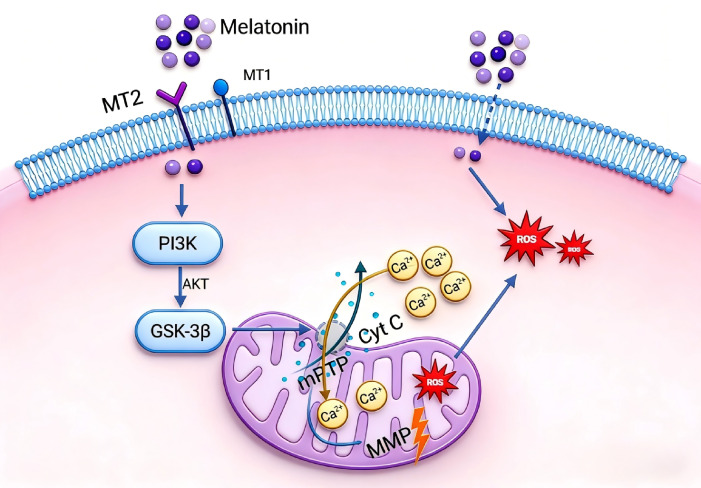
Schematic diagram of the antioxidant pathway of melatonin in frozen sperm of brown spot grouper: melatonin–MT2 receptor/PI3K/GSK-3β-mPTP-MMP.

Melatonin may improve the cryopreservation effect of brown-marbled grouper sperm through a potential dual mechanism: on the one hand, it penetrates the plasma membrane via its lipophilic property and transiently scavenges the reactive oxygen species (ROS) burst during the early stage of freezing–thawing, providing receptor-independent antioxidant protection; on the other hand, it may specifically activate the MT2 receptor and, based on pharmacological evidence, potentially inhibit the abnormal opening of mPTP through the PI3K/Akt/GSK-3β signaling axis, thereby maintaining mitochondrial membrane potential and motility (Figure 6). This study is the first to provide pharmacological evidence in the field of teleost sperm cryoprotection that the effects of melatonin may depend on the MT2 receptor, revealing potential differences from mammalian systems and filling a knowledge gap regarding the role of this signaling pathway in teleost reproductive biology. Meanwhile, this study provides a preliminary theoretical basis for the development of novel anti-cryodamage strategies targeting MT2/PI3K. The present study still has room for further improvement. At present, only pharmacological approaches were used to preliminarily elucidate the potential role of the related signaling pathway, and direct detection and verification have not been performed for key molecular events including PI3K activity, phosphorylation levels of Akt and GSK-3β, cytochrome c release, caspase activation, and apoptotic markers. In addition, systematic evaluation of fertilization rate has not been conducted in this study. Future research will directly verify key signaling molecules further, using molecular biological methods such as Western blot, flow cytometry, and enzyme activity assays. It will also combine fertilization and hatching experiments to improve the applied evaluation so as to more systematically and thoroughly clarify the molecular mechanism and application potential of melatonin in the cryopreservation of grouper sperm.

## Figures and Tables

**Table 1:** Information of specific inhibitors used in the experiment.

Inhibitor Name	Biological Function	Working Concentration
8-M-PDOT	Selective MT1 receptor inhibitor, MT2 receptor agonist	10^−6^ M
4-P-PDOT	Specific MT2 receptor antagonist	10^−6^ M
Luzindole	Broad-spectrum MT1/MT2 receptor antagonist	10^−6^ M
LY294002	Broad-spectrum PI3K inhibitor	10^−5^ M
SB415286	Specific GSK-3β inhibitor	10^−5^ M

Note: All inhibitors were incubated with sperm samples at 4 °C for 10 min for pre-cryopreservation equilibration before subsequent cryopreservation treatment.

## Data Availability

The original contributions presented in this study are included in the article. Further inquiries can be directed to the corresponding authors.

## Abbreviations

The following abbreviations are used in this manuscript:

MT	Melatonin
mPTP	Mitochondrial permeability transition pore
PI3K	Phosphatidylinositol 3-kinase
GSK-3β	Glycogen synthase kinase-3β
ROS	Reactive oxygen species
MMP	Mitochondrial membrane potential
CASA	Computer-Assisted Sperm Analysis
VCL	Curvilinear velocity
DMSO	Dimethyl sulfoxide
PBS	Phosphate-buffered saline
DCFH-DA	2′,7′-Dichlorodihydrofluorescein diacetate
JC-1	5,5′,6,6′-Tetrachloro-1,1′,3,3′-tetraethylbenzimidazolylcarbocyanine iodide
ANOVA	One-way analysis of variance
SD	Standard deviation
MDA	Malondialdehyde
Akt	Protein kinase B
SOD	Superoxide dismutase
CAT	Catalase
GPx	Glutathione peroxidase
AFMK	N1-Acetyl-N2-formyl-5-methoxykynuramine
Cytc	Cytochrome c
